# Risk factors for perioperative blood transfusion in patients undergoing total laparoscopic hysterectomy

**DOI:** 10.1186/s12905-024-02908-4

**Published:** 2024-01-24

**Authors:** Xianghua Cao, Xueliang Liu, Xingxing Zhang, Kefang Zhang, Chuan Chen, Qinfeng Yang, Jian Wang, Xueping Li, Ling Wei

**Affiliations:** 1 Department of Anesthesiology, Dongguan Tungwah Hospital, Dongguan, China; 2 Department of Obstetrics and Gynecology, Core Facility Center, The First Affiliated Hospital of USTC, Division of Life Sciences and Medicine, University of Science and Technology of China, Hefei, 230001 Anhui China; 3 Division of Orthopaedic Surgery, Department of Orthopaedics, Nanfang Hospital, Southern Medical University, Guangzhou, 510515 Guangdong China; 4 Nurse in Charge, Undergraduate, Nursing Department, People’s Hospital of Ganzhou, Ganzhou, China

**Keywords:** Blood transfusion, Total laparoscopic hysterectomy, Risk factors, Nationwide inpatient sample

## Abstract

**Purpose:**

The goal is to identify risk factors associated with receiving a blood transfusion during the perioperative period in patients who undergo total laparoscopic hysterectomy (TLH) using a large-scale national database.

**Methods:**

In this retrospective analysis, data from the Nationwide Inpatient Sample (NIS) was utilized to review the medical records of all patients who underwent TLH from 2010 to 2019. The researchers identified patients who had received a blood transfusion during the perioperative period and compared with those who had not. The subsequent factors associated with blood transfusion were examined: hospital characteristics (type of admission and payer, patient demographics (age and race), bed size, teaching status, location, and region of hospital), length of stay (LOS), total charges during hospitalization, in-hospital mortality, comorbidities, and perioperative complications. The data was analyzed using descriptive statistics. The independent risk factors of perioperative blood transfusion after TLH was identified by performing multivariate logistic regression.

**Results:**

A total of 79,933 TLH were captured from the NIS database, among which 3433 (4.40%) patients received a perioperative blood transfusion. TLH patients affected by blood transfusion were 2 days longer hospital stays (*P < 0.001*), higher overall costs (*P < 0.001*), the patients who received a transfusion after a long-term hospitalization had a significantly higher rate of mortality (0.5% vs. 0.1%; *P <* 0.001). Perioperative blood transfusion after TLH was associated with chronic blood loss anemia, deficiency anemia, coagulopathy, congestive heart failure, fluid and electrolyte disorders, renal failure, metastatic cancer, sepsis, weight loss, deep vein thrombosis, gastrointestinal hemorrhage, shock, acute myocardial infarction, and pneumonia, stroke, hemorrhage, pulmonary embolism, and disease of the genitourinary system.

**Conclusion:**

Studying the risk factors of perioperative blood transfusion after TLH is advantageous in order to ensure proper management and optimize outcomes.

**Supplementary Information:**

The online version contains supplementary material available at 10.1186/s12905-024-02908-4.

## Introduction

Hysterectomy is a commonly performed surgical procedure [1]. There are over 600,000 procedures conducted annually in the United States [2]. Laparoscopic hysterectomy (LH) has gained significant recognition as a favorable method of hysterectomy due to its link to relatively low rates of complications after surgery, minimal bleeding, have a brief hospitalization, and swiftly resume their regular activities [3, 4]. Although total laparoscopic hysterectomy (TLH) is generally safe, like any other surgical procedure, there is a potential risk of complications. It is worth mentioning that blood transfusions are commonly performed during surgeries. Although safety measures are in place nowadays, blood transfusions still carry risks, including acute reactions, medical mistakes, and the potential for viral or bacterial infections [5, 6]. Therefore, it is crucial to develop a strategy before surgery to prevent the need for perioperative blood transfusions in patients undergoing the TLH.

In recent years, several studies have reported some transfusion risk factors during hysterectomy. In a retrospective study, 517 patients were identified and included, revealing that 47 (9.09%) individuals received a blood transfusion during the perioperative period. It was discovered that pelvic adhesion increased the risk of receiving a blood transfusion for those undergoing TLH [5]. Another study discovered that patients who had a larger uterus, a greater estimated blood loss during the procedure, and had a longer operative time were found to be more prone to receiving a blood transfusion [7]. Early identification of patients at risk of requiring perioperative blood transfusions is widely recognized as a means to improve patient counseling, surgical preparation and blood protection, reduce blood transfusions related complications and effectively improve perioperative prognosis. However, until now, there is no study investigating the factors that increase the odds of requiring a blood transfusion undergoing TLH based on a large-scale sample database.

Consequently, we conducted a study aiming to detect the factors that increase the likelihood of perioperative blood transfusions in patients undergoing TLH using the Nationwide Inpatient Sample (NIS) database. We analyzed various factors that are related, such as patient demographics (age and race), characteristics of the hospital (type of admission and payer, bed size, teaching status, location, and region), length of stay (LOS), total charges during hospitalization, in-hospital mortality, comorbidities, and perioperative complications.

## Materials and methods

### Database

The most comprehensive all-payer healthcare database for inpatient hospital stays in the United States is the NIS database [8]. The NIS comprises hospital-discharge data at the patient level, provided by states participating in the Healthcare Cost and Utilization Project sponsored by the Agency for Healthcare Research and Quality [6, 9]. The NIS contains data elements regarding demographics, medical ailments, treatments, duration of hospitalization, and expenses [10].

### TLH procedure

A hysterectomy is the surgical removal of a woman’s uterus, and it is the frequently performed surgery solely in women [11]. A hysterectomy is typic usually performed in those with adenomyosis, leiomyoma, uterine prolapse, malignancy, and chronic bleeding [12]. Hysterectomy is generally carried out via the following three conventional approaches: abdominal, vaginal, and laparoscopic. In recent years, minimally invasive surgery has received more attention from surgeons because of increasing concerns regarding cosmetic outcomes of surgery [13]. TLH is performed with the intention of making abdominal hysterectomy a laparoscopic surgery [14]. Depending on the size of the uterus, a veress needle was placed either supraumbilically or through the umbilicus, and CO_2_ was then inhaled into the abdomen. Following the installation of the videolaparoscope and pneumoperitoneum, three suprapubic cannulas were inserted. After the procedure, a laparoscopy was used to reassess the pelvis and abdomen in order to perform pelvic lavage and ensure hemostasis [15]. Most studies set the indications for TLH as: usually less than 20 weeks’ gestation (below the navel level) [15], and no fertility requirement of adenomyosis, uterine fibroids, early endometrial cancer, etc. The uterus is enlarged beyond the umbilicus, severe cardiovascular disease, excessive obesity and severe uterine adhesions should be considered a contraindication for TLH. Depending on the size and position of the myomas, excessively enlarged uteri may limit access to the uterine vascular pedicles. They may also increase the risk of problems such bleeding, bowel and urine damage from inadequate exposure, difficulties removing the uterus, and prolonged surgery [15]. At present, most studies have modified the surgical methods to reduce the complications after TLH [16–18]. TLH is a Class 4 procedure that requires qualified physicians to perform the operation.

### Study population

**Fig. 1:**
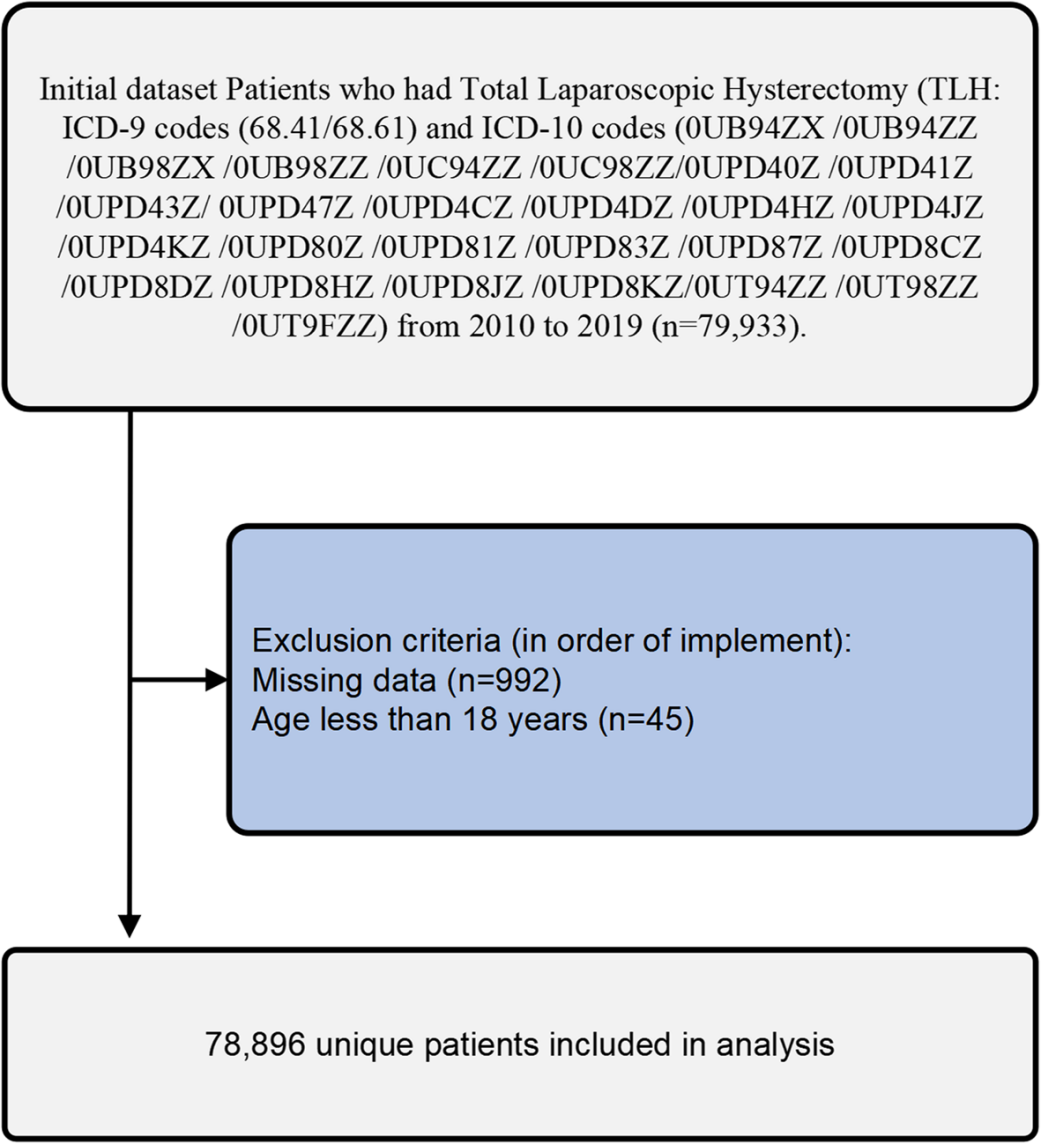
Flow and inclusion/exclusion of all patients had TLH

We conducted a retrospective analysis of individuals who had undergone TLH surgery from 2010 to 2019. We detected cases of TLH based on the occurrence of the ICD-9-CM procedure code 68.41, 68.61, and the ICD-10-CM procedure code 0UB94ZX /0UB94ZZ /0UB98ZX /0UB98ZZ /0UC94ZZ /0UC98ZZ/0UPD40Z /0UPD41Z /0UPD43Z/ 0UPD47Z /0UPD4CZ /0UPD4DZ /0UPD4HZ /0UPD4JZ /0UPD4KZ /0UPD80Z /0UPD81Z /0UPD83Z /0UPD87Z /0UPD8CZ /0UPD8DZ /0UPD8HZ /0UPD8JZ /0UPD8KZ/0UT94ZZ /0UT98ZZ /0UT9FZZ. Most of the missing values in this study were based on patient characteristics in the NIS database, and there were almost no missing data on comorbidities and complications, and any missing data were excluded in this study (Fig. 1). Patients younger than 18 years were also excluded. A total of 78,896 unique patients were included in the study. Because this study relied on data that is accessible to the public, institutional review board approval was deemed unnecessary, as stated in the Tri-Council Policy statement (2010).

### Outcomes

Covariates in the statistical models consisted of factors such as patient demographics, hospital characteristics, patient comorbidities, and postoperative complications, all of which were associated with blood transfusions. In our study, about 20% of records did not include information about the patients race and/or ethnicity. Because some states do not document the race variable on the discharge records to protect patient confidentiality [10]. In order to deal with this problem, we implemented a new category called “others” to record the racial information of these patients. The categorization of insurance status included options such as Medicare, Medicaid, private insurance, lack of insurance, or other. Hospital attributes consisted of geographical region, bed count, teaching status, and whether it was located in an urban or rural area. The presence of 29 different comorbidities was identified by classifying them using the Agency for Healthcare Research and Quality Comorbidity Software, version 3.7. The aforementioned comorbidities are flagged in this software using ICD-9-CM and ICD-10-CM diagnosis codes.

### Statistical analysis

We began by examining the data distribution and calculating the basic descriptive statistics for all the variables included in the analysis. Afterwards, the continuous variable data were compared using a T-test, while the categorical data were compared using either χ2 test or Fisher exact test. To identify the factors that independently predict blood transfusion after TLH, we conducted univariate and multivariate regression analyses. In order to evaluate the impact of various pre-surgery factors on the requirement for blood transfusions, we computed the adjusted odds ratio. These factors included patient characteristics like age, race, and teaching status of hospitals, as well as patient comorbidities and concurrent operations such as wound infection, hemorrhage, and pulmonary embolism. We used SPSS 25 (IBM, USA) to carry out all statistical analyses on the NIS database. A *P*-value less than 0.05 was considered statistically significant.

## Results

### Transfusion trends

**Fig. 2:**
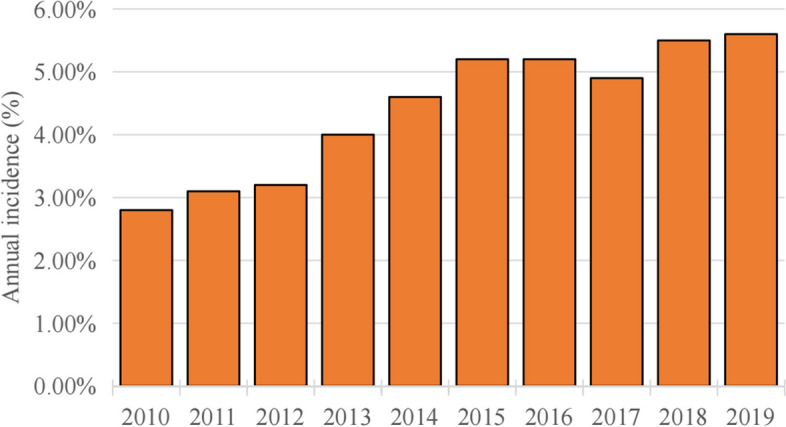
Annual incidence of blood transfusion after TLH

Patient demographics, hospital characteristics, and comorbidities are among the categories of variables outlined in Table 1. Patient characteristics and outcomes were showed Table 2. During the period from 2010 to 2019, a total of 79,933 patients in the United States underwent TLH. Of which,78,896 were identified and included in the analysis (Fig. 1). Out of a total of 3433 patients, 4.40% underwent a blood transfusion during the perioperative period. The blood transfusions rates varied between 2.8% in 2010 and 5.6% in 2019 (Fig. 2). There was a slight yearly increase in the rate of blood transfusions.

**Table 1:** Variables used in binary logistic regression analysis

Variables Categories	Specific Variables
Patient demographics	Age (≤64 years and ≥ 65 years), race (White, Black, Hispanic, Asian or Pacific Islander, Native American and Other)
Hospital characteristics	Type of admission (non-elective, elective), bed size of hospital (small, medium, large), teaching status of hospital (nonteaching, teaching), location of hospital (rural, urban), type of insurance (Medicare, Medicaid, private insurance, self-pay, no charge, other), location of the hospital (northeast, Midwest or north central, south, west)
Comorbidities	AIDS, alcohol abuse, deficiency anemia, rheumatoid diseases, chronic blood loss anemia, congestive heart failure, chronic pulmonary disease, coagulopathy, depression, diabetes (uncomplicated), diabetes (with chronic complications), drug abuse, hypertension, hypothyroidism, liver disease, lymphoma, fluid and electrolyte disorders, metastatic cancer, neurological disorders, obesity, paralysis, peripheral vascular disorders, psychoses, pulmonary circulation disorders, renal failure, solid tumor without metastasis, peptic ulcer disease, valvular disease and weight loss

*AIDS* Acquired immunodeficiency syndrome

**Table 2:** Patient characteristics and outcomes after total laparoscopic hysterectomy (2010–2019)

Characteristics	Transfusion	No Transfusion	P
Total (n = count)	3433	75,463	
Total incidence (%)	4.40		
Age (median, years)	47 (41–57)	48 (42–61)	< 0.001
Age group n (%)			
18–44	1398 (40.7)	26,835 (35.60)	< 0.001
45–64	1457 (42.40)	34,467 (45.70)	
65–74	330 (9.6)	9653 (12.8)	
≥75	248 (7.2)	4508 (6.0)	
Race n (%)			
White	1531 (44.60)	45,540 (60.30)	< 0.001
Black	827 (24.10)	9538 (12.60)	
Hispanic	536 (15.60)	9930 (13.20)	
Asian or Pacific Islander	214 (6.20)	2496(3.30)	
Native American	12 (0.30)	369 (0.50)	
Other	313 (9.1)	7590 (10.1)	
Number of Comorbidity n (%)			
0	683 (19.90)	25,212 (33.40)	< 0.001
1	950 (27.70)	20,453 (27.10)	
2	643 (18.70)	13,851 (18.40)	
≥3	1157 (33.70)	15,947 (21.10)	
LOS (median, d)	3 (2–5)	1 (1–2)	< 0.001
TOTCHG (median, $)	58,766 (38,001-95,386)	40,922 (26,374-61,993)	< 0.001
Type of insure n (%)			
Medicare	686 (20.00)	16,032 (21.20)	< 0.001
Medicaid	663 (19.30)	10,080 (13.40)	
Private insurance	1769 (51.50)	44,860 (59.40)	
Self-pay	192 (5.60)	1676 (2.20)	
No charge	35 (1.00)	354 (0.50)	
Other	88 (2.60)	2461 (3.30)	
Bedsize of hospital n (%)			
Small	468 (13.60)	9562 (12.70)	0.017
Medium	1021(29.70)	21,335 (28.30)	
Large	1944 (56.60)	44,566 (59.10)	
Elective admission n (%)	2090 (60.90)	66,550 (88.20)	< 0.001
Type of hospital, teaching n (%)	2411 (70.20)	51,250 (67.90)	0.004
Location of hospital, urban n (%)	3248 (94.60)	71,643 (94.90)	0.394
Region of hospital n (%)			
Northeast	645 (18.80)	14,739 (19.50)	< 0.001
Midwest or North Central	514 (15.00)	15,051(19.90)	
South	1479 (43.10)	25,270 (33.50)	
West	795 (23.20)	20,403 (27.00)	
Died n (%)	16 (0.50)	58 (0.10)	< 0.001

*LOS* Length of stay: *TOTCHE* Total charge

Bedsize assesses the number of short-term acute beds in a hospital. The hospital’s bedsize category is nested within location and teaching status. The Medicare group consisted of both fee-for-service and managed care for Medicare patients, Federal health insurance for those aged 65+ and certain disabilities. The Medicaid group included both fee-for-service and managed care for Medicaid patients, Joint federal-state health coverage for low-income individuals. Private insurance: Employer-based or individually purchased health plans. Self-pay: No health coverage, paying out of pocket [35]

### Demographics

Both groups were no difference in terms of location of hospital (Table 2); In contrast, the blood transfusion group had a notably higher number of patients for all the analyzed patient characteristics and outcomes. TLH patients affected by blood transfusion were 1 year younger (47 years vs. 48 years, *P < 0.001*) (Table 2); TLH patients affected by blood transfusions were 2 days longer hospital stays (3 days vs. 1 day, *P < 0.001*) (Table 2), and higher overall costs ($58,766 vs. $40,922, *P < 0.001*) (Table 2)*;* Patients with blood transfusions are less likely to have elective admissions (60.9% vs. 88.2%, *P < 0.001*)*.* (Table 2); Additionally, blood transfusions after TLH tended to occur in non-teaching hospital (70.2% vs. 67.9%, *P < 0.001)* and people with comorbidities (*P < 0.001*) (Table 2)*.* As expected, in-hospital mortality of patients with transfusions after TLH was significantly higher, exceeding five times than patients with no transfusion (0.5% vs. 0.1%; *P <* 0.001) (Table 2)*.*

### Factors that increase the likelihood of experiencing a blood transfusion following TLH

**Fig. 3:**
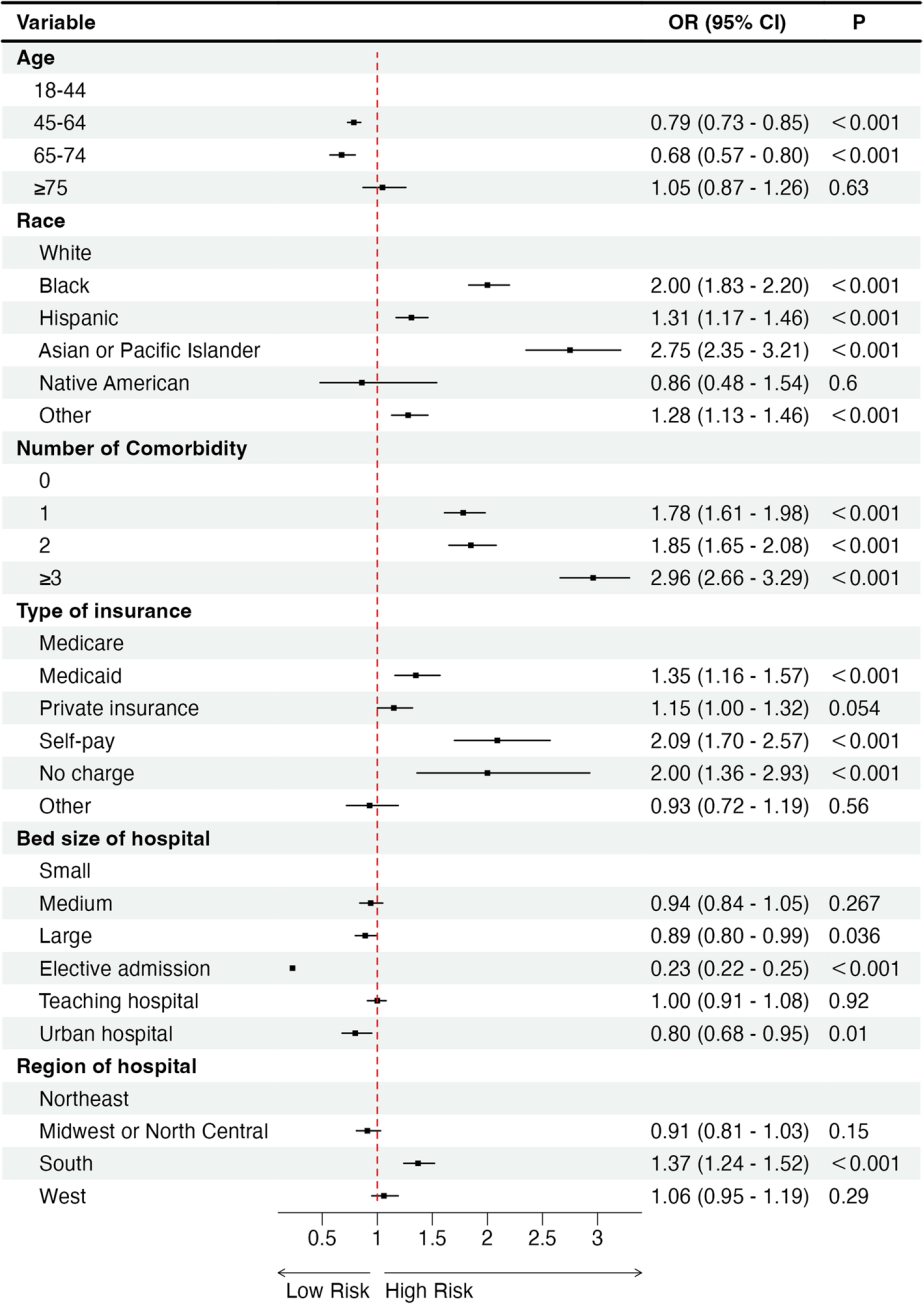
Risk factors associated with blood transfusion after TLH

A logistic regression analysis was conducted in order to determine the risk factors associated with transfusion post TLH (as seen in Table S1). The following factors were identified: race of black (odds ratio [OR] = 2.00; CI =1.83–2.20), race of hispanic (OR = 1.31; CI = 1.17–1.46), race of Aisan of pacific islander (OR = 2.75; CI = 2.35–3.21) (Table 3 and Fig. 3); 1 of comorbidity (OR = 1.78; CI = 1.61–1.98), 2 of comorbidity (OR = 1.85; CI = 1.65–2.08), 3 of comorbidity (OR = 2.96; CI = 2.66–3.29) (Table S1 and Fig. 3); medicaid type of insurance (OR = 1.35; CI = 1.16–1.57), self-pay type of insurance (OR = 2.09; CI = 1.70–2.57), no charge type of insurance (OR = 2.00; CI = 1.36–2.93) (Table 3 and Fig. 3); The south region of the hospital (OR = 1.37,CI = 1.24–1.52) and undergoing total laparoscopic hysterectomy were identified as independent risk factors for perioperative blood transfusions (*P < 0.001*).(Table S1 and Fig. 3).

### Comorbidities associated with blood transfusion following TLH

**Fig. 4:**
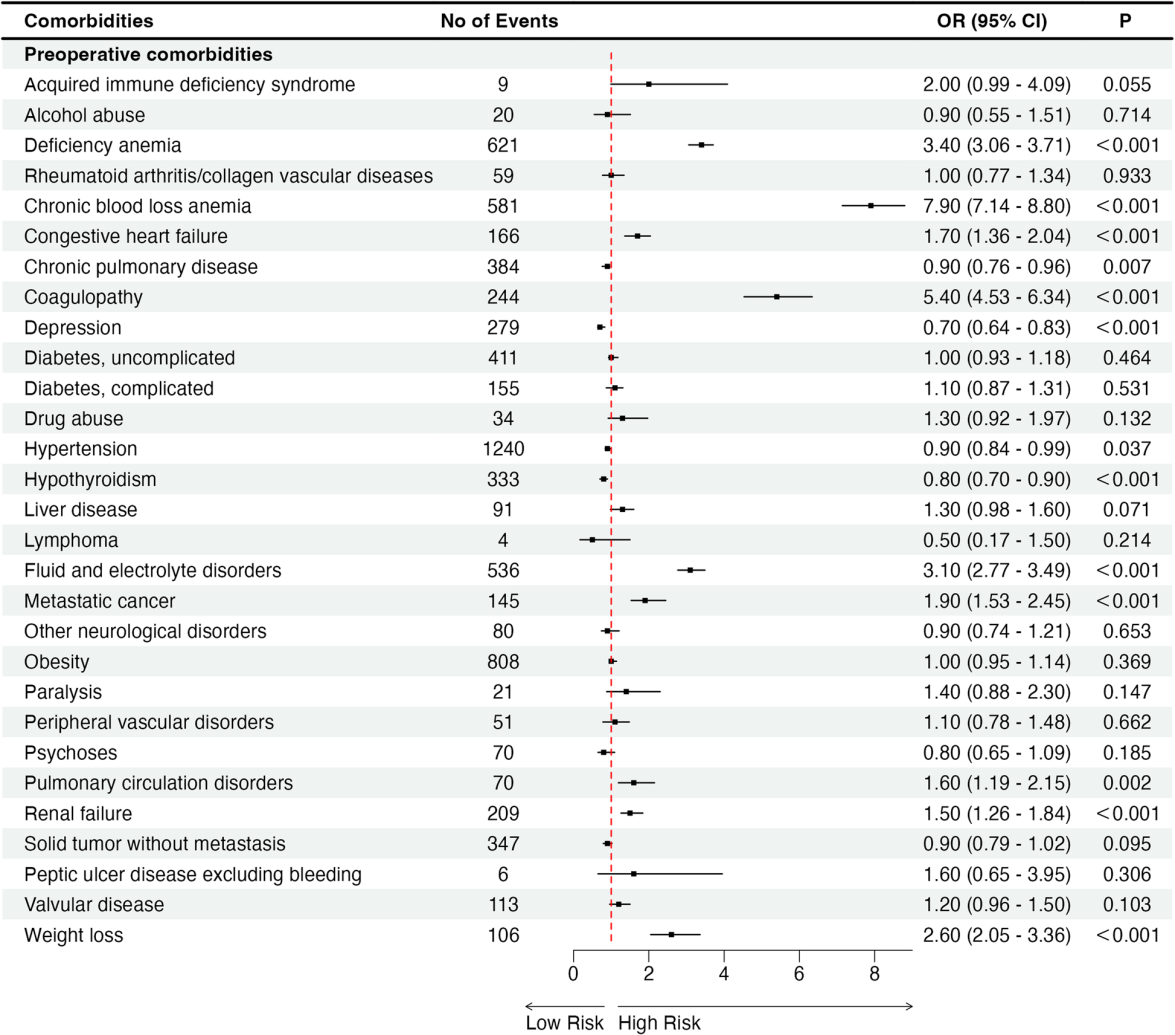
Comorbidities associated with blood transfusion following TLH

Regarding the indications for blood transfusion, the probability of perioperative blood transfusion during TLH is relatively low. However, the analysis of a single variable indicated that patients who had deficiency anemia, chronic blood loss anemia, congestive heart failure, coagulopathy, depression, fluid and electrolyte disorders, metastatic cancer, pulmonary circulation disorders, weight loss, valvular disease, peripheral vascular disease, obesity, liver disease, or diabetes with complicated or renal failure and were undergoing TLH were at a higher risk of needing a blood transfusion during the perioperative period (*P < 0.001*) (Table S2). In multivariate analysis, deficiency anemia (OR = 3.4; CI = 3.06–3.71), chronic blood loss anemia (OR = 7.9; CI = 7.14–8.80), congestive heart failure (OR = 1.7; CI = 1.36–2.04), coagulopathy (OR = 5.4; CI = 4.53–6.34), fluid and electrolyte disorders (OR = 3.1; CI = 2.77–3.49), metastatic cancer (OR = 1.9; CI = 1.53–2.45), renal failure (OR = 1.5; CI = 1.26–1.84) and weight loss (OR = 2.6; CI = 2.05–3.36) were independent risk factors for perioperative blood transfusion during TLH (*P < 0.001*) (Table S2 and Fig. 4).

### Complications associated with blood transfusion following TLH

**Fig. 5:**
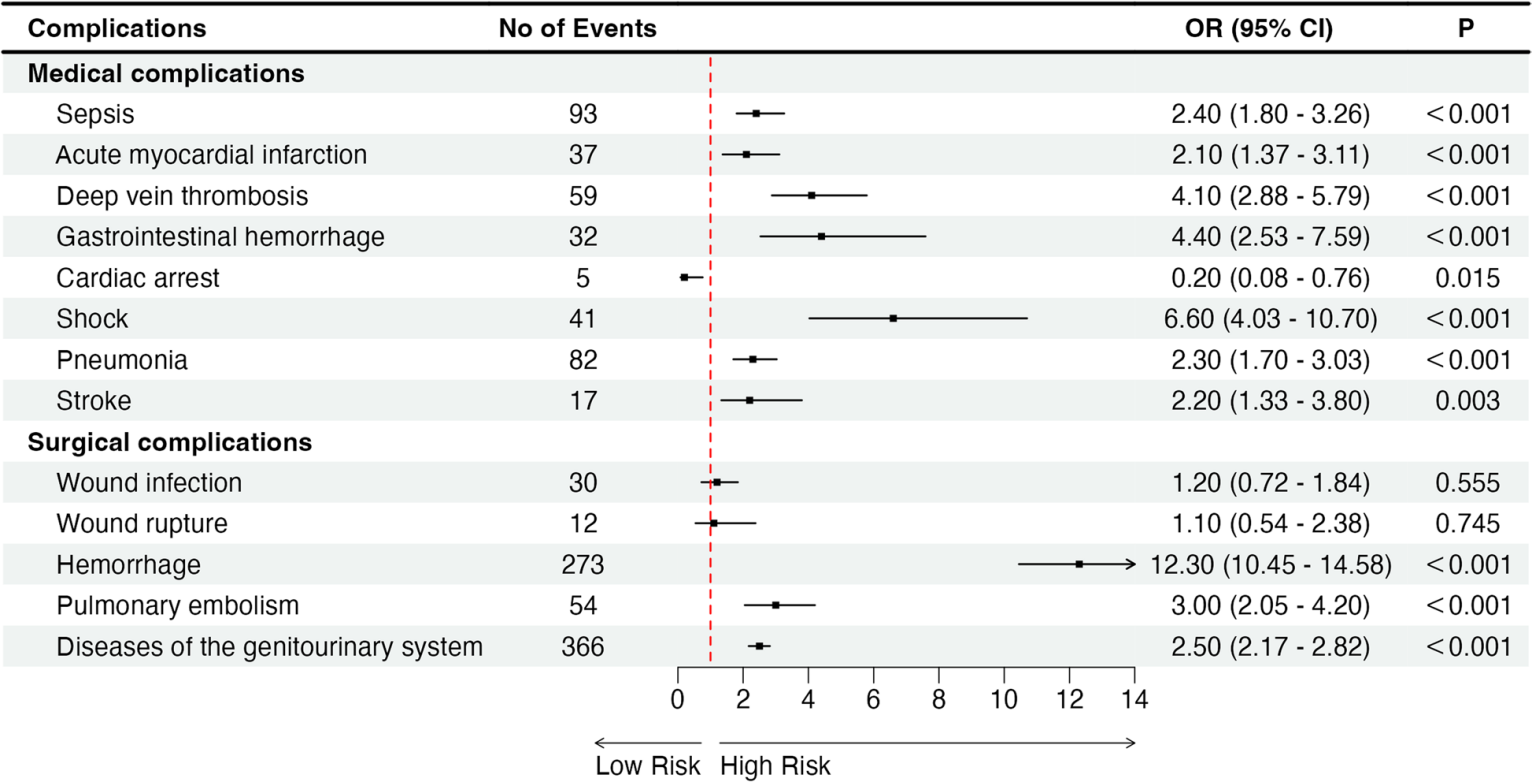
Complications associated with blood transfusion following TLH

Patients who had received blood transfusion were found to be at a higher risk of experiencing medical perioperative complications such as sepsis, acute myocardial infarction, deep vein thrombosis, gastrointestinal bleeding, shock, pneumonia, stroke, as well as surgical perioperative complications including wound infection, wound rupture, hemorrhage, pulmonary embolism, and genitourinary system diseases, when compared to patients who did not receive blood transfusion (*P < 0.001*) (Table S3). Multivariate analysis showed that in perioperative blood transfusion during TLH was independently associated with sepsis (OR = 2.4; CI = 1.80–3.26, *P < 0.001*), acute myocardial infarction (OR = 2.1; CI = 1.37–3.11, *P < 0.001*), deep vein thrombosis (OR = 4.1; CI = 2.88–5.79, *P < 0.001*), gastrointestinal hemorrhage (OR = 4.4; CI = 2.53–7.59, *P < 0.001*), shock (OR = 6.6; CI = 4.03–10.70, *P < 0.001*), and pneumonia (OR = 2.3; CI = 1.70–3.03, *P < 0.001*), stroke (OR = 2.2; CI = 1.33–3.80, *P = 0.003*), hemorrhage (OR = 12.3; CI = 10.45–14.58, *P < 0.001*), pulmonary embolism (OR = 3.0; CI = 2.05–4.20, *P < 0.001*), and disease of the genitourinary system (OR = 2.5; CI = 2.17–2.82, *P < 0.001*) (Table S3 and Fig. 5).

## Discussion

We conducted a study to examine the factors that contribute to blood transfusion during hospitalization in the United States. Among the total patient population, 3433 individuals (4.40%) underwent a blood transfusion during the perioperative period. The percentage of patients receiving blood transfusion varied from 2.8% in 2010 to 5.6% in 2019 (Fig. 2). It is worth noting that the rate of blood transfusion showed a gradual rise over the years. This increase may be partially due to the transition from allogenic transfusion to autologous (acute normovolemic hemodilution, intraoperative and postoperative autotransfusion) with advances in blood bank screening and storage [19, 20], as evident from the arise in autologous transfusion rates over the same time period. Dilutive autotransfusion and acute hemodilution have also been recorded as blood transfusions in clinical practice. This rise in numbers could also be attributed to recent improved documentation and coding practices [6, 21]. Because previous studies had indicated that the ICD-9 codes used for tracking blood transfusions did not accurately reflect the actual usage of blood products [6, 20].

There is no study investigating the transfusion trends in TLH at a national level. From 2018 to 2019, a retrospective study analyzed 517 patients who underwent hysterectomy for benign disease at a teaching institution. The study aimed to identify risk factors associated with perioperative blood transfusion. The findings revealed an overall blood transfusion rate of 9.09% [5], indicating a higher likelihood of needing a blood transfusion in a teaching institution. That may be because the situation of patients in teaching hospitals is more serious. In our study, the variable of teaching hospitals was also included for observation. The number of the retrospective studies is relatively small, and the observation time is relatively short [5]. It is possible that our study was more convincing to detect a the overall transfusion rate of TLH. One of the benefits of this study is the inclusion of both ICD-9 codes and ICD-10 codes, allowing for a comprehensive and extensive analysis of the health and economic aspects of peri-operative blood transfusion during TLH.

The risk of transfusion is influenced by factors related to demographics and hospitals, and this study’s logistic regression analysis provides valuable understanding of this correlation. As stated before, the study found that black patients are more likely to need a transfusion, which is likely attributed to the higher prevalence of anemia among this group [6, 22]. The research conducted by our team revealed notable variances in the heightened likelihood of requiring a transfusion among patients of African descent. It is important to acknowledge that urban hospitals in the Southern region of the United States have a higher likelihood of encountering complications during transfusion treatments. This disparity may be attributed to the variations in hospital standards and practices regarding transfusions, such as the criteria used to determine when a transfusion is necessary [6]. The group of no charge or self-pay might be unaffordable insurance, lower economic levels and poor health conditions (dietary iron deficiency, hemoglobinopathy, nutritional deficiencies, malaria and HIV/AIDS), which increase the need for blood transfusions [23]. Patients with comorbidities(≥1 of comorbidities) are more likely to require blood transfusions, especially elderly patients with other diseases. An observational study found that patients with endometrial Cancer are characterized by a high burden of comorbidities [24]. The surgical method for endometrial cancer is laparoscopic total hysterectomy. Further attention is needed to conduct study on elderly patients with other comorbidities.

Patients who have deficiency anemias, chronic anemia caused by blood loss, gastrointestinal bleeding, shock, hemorrhages, or coagulopathies are at the greatest risk of needing transfusions due to their low hemoglobin levels and tendency to bleed [5, 6]. Deficiency anemia is classified as either iron deficiency anemia, vitamin deficiency anemia (B12 or folic acid), protein deficiency anemia, or anemia of chronic disease (such as chronic kidney disease) [25]. In our study, we confirmed this with a large sample database. And, our research further found that patients with fluid and electrolyte disorders, metastatic cancer, renal failure, or weight loss carry the highest risks of requiring transfusions probably due to the iron deficiency anemia, vitamin deficiency anemia (B12 or folic acid), protein deficiency anemia, nutrient loss or anemia of chronic disease.

In line with previous research [8, 25–28], our data analysis revealed that patients who received peri-operative blood transfusion had a more extended average hospital stay (around 2 days longer) and increased higher hospital costs than those who did not receive transfusion. The findings of our study indicate poorer outcomes in patients who underwent blood transfusion. Nevertheless, it is uncertain whether the blood transfusions result or contribute to these unfavorable outcomes in these patients. Transfusion risk for sepsis was greater significantly than that of wound infection. Probably because sepsis is more severe, with greater circulatory dysfunction, greater susceptibility to anemia and blood transfusions. According to clinical experience, sepsis occurs after the wound becomes infected.

Multiple research studies have demonstrated a link between blood transfusion and increased rates of mortality and complications following various surgical procedures [26–33]. From 1996 to 1997, 9482 patients were prospectively evaluated on the impact of blood management funds on patients receiving allogenic blood products. According to the study’s results, these patients were found to have a higher susceptibility to infections, deep vein thrombosis, and fluid overload [33]. The research, which involved 9598 individuals diagnosed with consecutive hip fractures, revealed a 35% increased likelihood of experiencing severe bacterial infections and a 52% increased likelihood of developing pneumonia [32].

The results of our investigation confirmed that there was an increase in both surgical and medical complications as a result of blood transfusion. Specifically, patients who underwent a blood transfusion had a significantly increased risk of developing postoperative infections, pulmonary insufficiency, pulmonary edema, and venous thromboembolism. There have been observational studies that have shown a connection between receiving a blood transfusion and experiencing a higher probability of postoperative infections, a lengthier period of time staying in the hospital, and an increased likelihood of mortality in patients who are undergoing non-cardiac surgery [34], and orthopaedic surgery [27, 32, 33]. Our study found that patients who were given a transfusion had consistently higher mortality rates during their hospital stay compared to those who did not receive a transfusion.

This study’s limitations primarily stem from analyzing secondary data from extensive administrative databases. Firstly, The NIS does not include clinical information such as the type of anesthesia used, the amount of blood loss, the hemoglobin values during the perioperative period, and the quantity or volume of blood units transfused [6]. This is the weakness of this study, but this does not affect the analysis of risk factors. The NIS database has the advantage of a relatively large sample size and the data was more convincing. The results of this study provide better treatment strategies for the development of women’s health. Secondly, The NIS database is an inpatient database that only captures information on early complications during hospitalization, not variables for long-term complications after discharge. Due to the absence of outpatient follow-up, the NIS database solely captures in-patient data. Consequently, readmissions and post discharge complications such as blood transfusions cannot be documented [8]. Incomplete or incorrect coding of blood-product administration and surgical complications may occur. The accuracy of our calculations for blood transfusions depends on the precision and reliability of the codes we use. Nevertheless, it is important to provide additional information regarding the accuracy and effectiveness of these codes [6]. Thirdly, while we managed to achieve equilibrium among various groups based on identifiable variables, we cannot fully consider hidden variables that may influence the results, which is a fundamental constraint of all observational studies. TLH was selected as the main observation variable in our study. It is not strictly defined some surgical indications (endometrial cancer, abnormal uterine bleeding, uterine intracavitary pathologies, etc). The NIS database also does not have records of surgical duration, uterine and fibroid size. There may be some degree of bias, but due to the large sample size we observed, this does not affect the results of this study. In the future, we will conduct some prospective studies to observe the incidence and specific risk factors of perioperative blood transfusion for some diseases relating to TLH. Finally, interpreting the impact of race as a risk factor for transfusion requires exercising caution, considering that approximately 20% of the data on this factor is unavailable.

## Conclusion

In conclusion, the healthcare systems are worried about the increasing frequency of blood transfusions and how it affects patients undergoing TLH. Women (black, hispanic, asian or pacific islander; number of comorbidity ≥1; self-pay of insurance, no charge of insurance; south region of hospital; chronic blood loss anemia, deficiency anemia, metastatic cancer, and coagulopathy, fluid and electrolyte disorders, renal failure, weight loss; sepsis, acute myocardial infarction, deep vein thrombosis, gastrointestinal hemorrhage, shock, Pneumonia, hemorrhage, pulmonary embolism, diseases of the genitourinary system) with higher chances of needing a perioperative blood transfusion (Fig. S1). These patients should receive appropriate counseling, blood protection method and sufficient preparation for surgery. This will help prevent unnecessary interventions during the surgical procedure.

In the future, it is necessary to conduct further large-scale, multicenter prospective studies to observe some diseases relating to TLH and long-term complications related to blood transfusion.

### Supplementary Information

**Additional file 1:** **Figure S1.** Overview of risk factors associated with blood transfusion after TLH

**Additional file 2:** **Table S1.** Risk factors associated with blood transfusion after TLH

**Additional file 3:** **Table S2.** Relationship between blood transfusion and preoperative comorbidities

**Additional file 4:** **Table S3.** Relationship between blood transfusion and postoperative complications

## Data Availability

This study is based on data provided by Nationwide Inpatient Sample (NIS) database, part of the Healthcare Cost and Utilization Project, Agency for Healthcare Research and Quality. The NIS database is a large publicly available full-payer inpatient care database in the United States and the direct web link to the database is https://www.ahrq.gov/data/hcup/index.html. Therefore, individual or grouped data cannot be shared by the authors.

## Abbreviations

TLH: Total laparoscopic hysterectomy
NIS: Nationwide Inpatient Sample
LOS: Length of stay
OR: Odds ratio
95% CI: 95% confidence interval
