# Supplementary figures and images for: Risk factors for perioperative blood transfusion in patients undergoing total laparoscopic hysterectomy
Source: BMC Womens Health. 2024 Jan 24;24:65. doi: 10.1186/s12905-024-02908-4 (PMC10809697; doi:10.1186/s12905-024-02908-4)

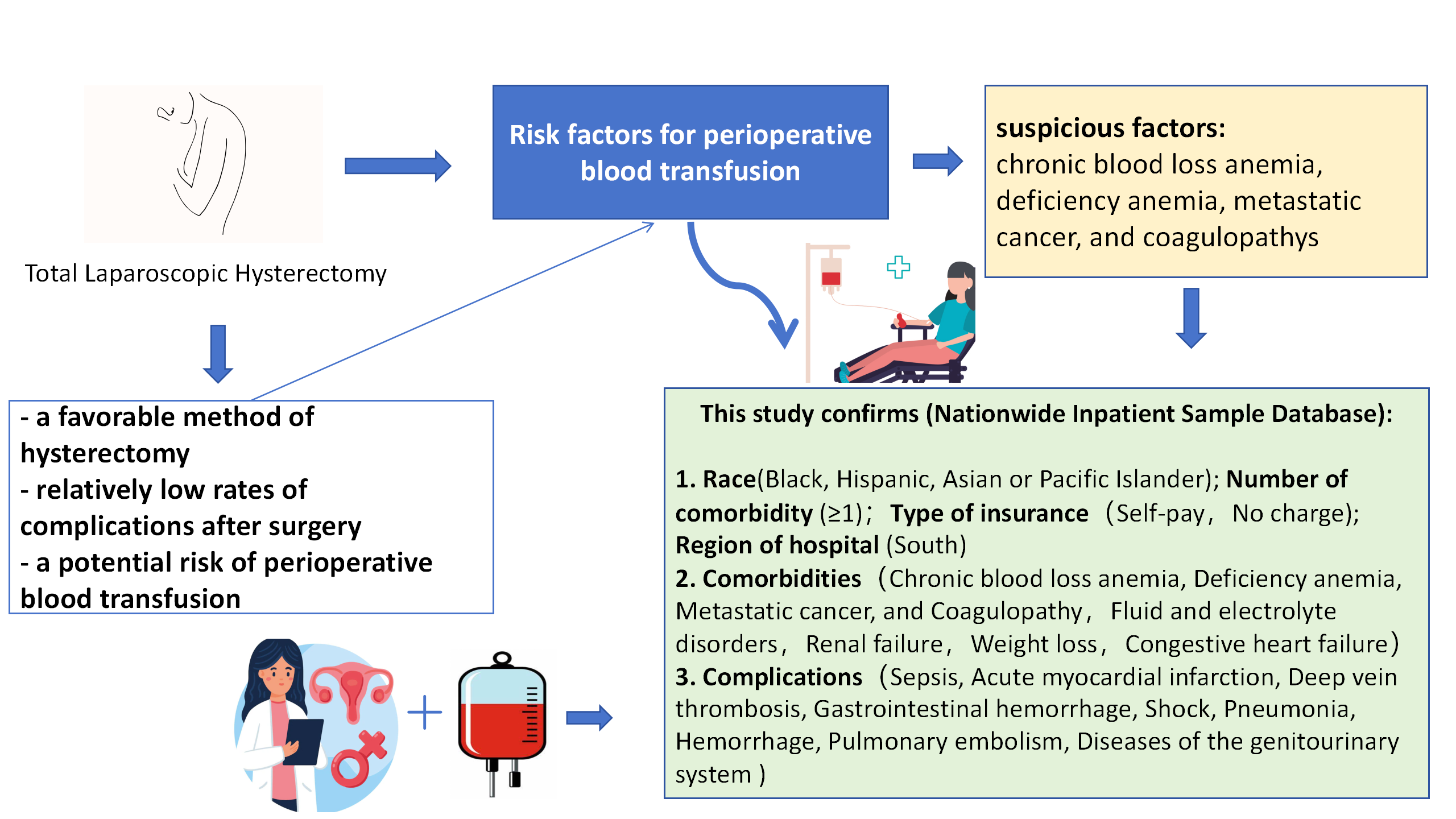

Supplement: Supplementary file 1 — Additional file 1: Figure S1. Overview of risk factors associated with blood transfusion after TLH [file 12905_2024_2908_MOESM1_ESM.tif]
