# Supplementary material for: Risk factors for perioperative blood transfusion in patients undergoing total laparoscopic hysterectomy
Source: BMC Womens Health. 2024 Jan 24;24:65. doi: 10.1186/s12905-024-02908-4 (PMC10809697; doi:10.1186/s12905-024-02908-4)
Supplement: Supplementary file 2 — Additional file 2: Table S1. Risk factors associated with blood transfusion after TLH [file 12905_2024_2908_MOESM2_ESM.docx]

**Table S1** Risk factors associated with blood transfusion after Total laparoscopic hysterectomy

| **Variable** | | **Multivariate Logistic Regression** | | |
| --- | --- | --- | --- | --- |
|  |  | **OR** | **95% CI** | **P** |
| **Age** | |  |  |  |
| 18-44 | | Ref | —— | —— |
| 45-64 | | 0.787 | 0.73-0.85 | ＜0.001 |
| 65-74 | | 0.676 | 0.57-0.80 | ＜0.001 |
| ≥75 | | 1.047 | 0.87-1.26 | 0.630 |
| **Race** | |  |  |  |
|  | White | Ref | —— | —— |
|  | Black | 2.00 | 1.83-2.20 | ＜0.001 |
|  | Hispanic | 1.31 | 1.17-1.46 | ＜0.001 |
|  | Asian or Pacific Islander | 2.75 | 2.35-3.21 | ＜0.001 |
|  | Native American | 0.86 | 0.48-1.54 | 0.60 |
|  | Other | 1.28 | 1.13-1.46 | ＜0.001 |
| **Number of Comorbidity** | |  |  |  |
|  | 0 | Ref | —— | —— |
|  | 1 | 1.78 | 1.61-1.98 | ＜0.001 |
|  | 2 | 1.85 | 1.65-2.08 | ＜0.001 |
|  | ≥3 | 2.96 | 2.66-3.29 | ＜0.001 |
| **Type of insurance** | |  |  |  |
|  | Medicare | Ref | —— | —— |
|  | Medicaid | 1.35 | 1.16-1.57 | ＜0.001 |
|  | Private insurance | 1.15 | 1.00-1.32 | 0.054 |
|  | Self-pay | 2.09 | 1.70-2.57 | ＜0.001 |
|  | No charge | 2.00 | 1.36-2.93 | ＜0.001 |
|  | Other | 0.93 | 0.72-1.19 | 0.560 |
| **Bed size of hospital** | |  |  |  |
|  | Small | Ref | —— | —— |
|  | Medium | 0.94 | 0.84-1.05 | 0.267 |
|  | Large | 0.89 | 0.80-0.99 | 0.036 |
| **Elective admission** | | 0.23 | 0.22-0.25 | ＜0.001 |
| **Teaching hospital** | | 1.00 | 0.91-1.08 | 0.92 |
| **Urban hospital** | | 0.80 | 0.68-0.95 | 0.01 |
| **Region of hospital** | |  |  |  |
|  | Northeast | Ref | —— | —— |
|  | Midwest or North Central | 0.91 | 0.81-1.03 | 0.15 |
|  | South | 1.37 | 1.24-1.52 | ＜0.001 |
|  | West | 1.06 | 0.95-1.19 | 0.29 |

AIDS: Acquired immunodeficiency syndrome, OR: Odds ratio, CI: Confidence interval
