# Supplementary material for: Risk factors for perioperative blood transfusion in patients undergoing total laparoscopic hysterectomy
Source: BMC Womens Health. 2024 Jan 24;24:65. doi: 10.1186/s12905-024-02908-4 (PMC10809697; doi:10.1186/s12905-024-02908-4)
Supplement: Supplementary file 3 — Additional file 3: Table S2. Relationship between blood transfusion and preoperative comorbidities [file 12905_2024_2908_MOESM3_ESM.docx]

**Table S2** Relationship between blood transfusion and preoperative comorbidities

| **Comorbidities** | | **Univariate Analysis** | | | **Multivariate Logistic Regression** | | | |
| --- | --- | --- | --- | --- | --- | --- | --- | --- |
|  |  | **No transfusion** | **Transfusion** | **P** | | **OR** | **95% CI** | **P** |
| **Preoperative comorbidities** | |  |  |  | |  |  |  |
|  | Acquired immune deficiency syndrome | 99 (0.1%) | 9 (0.3%) | 0.042 | | 2.0 | 0.99-4.09 | 0.055 |
|  | Alcohol abuse | 290 (0.4%) | 20 (0.6%) | 0.069 | | 0.9 | 0.55-1.51 | 0.714 |
|  | Deficiency anemia | 4,926 (6.5%) | 621 (18.1%) | ＜0.001 | | 3.4 | 3.06-3.71 | *＜0.001* |
|  | Rheumatoid arthritis/collagen vascular diseases | 1,170 (2.8%) | 59 (1.7%) | 0.436 | | 1.0 | 0.77-1.34 | 0.933 |
|  | Chronic blood loss anemia | 2,123 (2.8%) | 581 (16.9%) | ＜0.001 | | 7.9 | 7.14-8.80 | *＜0.001* |
|  | Congestive heart failure | 1,137 (1.5%) | 166 (4.8%) | ＜0.001 | | 1.7 | 1.36-2.04 | *＜0.001* |
|  | Chronic pulmonary disease | 8,798 (11.7%) | 384 (11.2%) | 0.398 | | 0.9 | 0.76-0.96 | *0.007* |
|  | Coagulopathy | 724 (1.0%) | 244 (7.1%) | ＜0.001 | | 5.4 | 4.53-6.34 | ＜0.001 |
|  | Depression | 7,765 (10.3%) | 279 (8.1%) | ＜0.001 | | 0.7 | 0.64-0.83 | ＜0.001 |
|  | Diabetes, uncomplicated | 8,491 (11.3%) | 411 (12.0%) | 0.192 | | 1.0 | 0.93-1.18 | 0.464 |
|  | Diabetes, complicated | 1,753(2.3%) | 155(4.5%) | ＜0.001 | | 1.1 | 0.87-1.31 | 0.531 |
|  | Drug abuse | 444 (0.6%) | 34 (1.0%) | 0.003 | | 1.3 | 0.92-1.97 | 0.132 |
|  | Hypertension | 25,676(34.0%) | 1,240 (36.1%) | 0.011 | | 0.9 | 0.84-0.99 | *0.037* |
|  | Hypothyroidism | 8,704 (11.5%) | 333(9.7%) | 0.001 | | 0.8 | 0.70-0.90 | *＜0.001* |
|  | Liver disease | 1,050 (1.4%) | 91 (2.7%) | ＜0.001 | | 1.3 | 0.98-1.60 | 0.071 |
|  | Lymphoma | 112 (0.1%) | 4 (0.1%) | 0.633 | | 0.5 | 0.17-1.50 | 0.214 |
|  | Fluid and electrolyte disorders | 2,868 (3.8%) | 536 (15.6%) | ＜0.001 | | 3.1 | 2.77-3.49 | *＜0.001* |
|  | Metastatic cancer | 1,640 (2.2%) | 145 (4.2%) | ＜0.001 | | 1.9 | 1.53-2.45 | *＜0.001* |
|  | Other neurological disorders | 1,304 (1.7%) | 80 (2.3%) | 0.009 | | 0.9 | 0.74-1.21 | 0.653 |
|  | Obesity | 15,851 (21%) | 808 (23.5%) | ＜0.001 | | 1.0 | 0.95-1.14 | 0.369 |
|  | Paralysis | 238 (0.3%) | 21 (0.6%) | 0.003 | | 1.4 | 0.88-2.30 | 0.147 |
|  | Peripheral vascular disorders | 633 (0.8%) | 51 (1.5%) | ＜0.001 | | 1.1 | 0.78-1.48 | 0.662 |
|  | Psychoses | 1,590 (2.1%) | 70(2.0%) | 0.786 | | 0.8 | 0.65-1.09 | 0.185 |
|  | Pulmonary circulation disorders | 426 (0.6%) | 70 (2.0%) | ＜0.001 | | 1.6 | 1.19-2.15 | *0.002* |
|  | Renal failure | 1,648 (2.2%) | 209 (6.1%) | ＜0.001 | | 1.5 | 1.26-1.84 | *＜0.001* |
|  | Solid tumor without metastasis | 7,991 (10.6%) | 347 (10.1%) | 0.369 | | 0.9 | 0.79-1.02 | 0.095 |
|  | Peptic ulcer disease excluding bleeding | 58(0.1%) | 6(0.2%) | 0.096 | | 1.6 | 0.65-3.95 | 0.306 |
|  | Valvular disease | 1,401 (1.9%) | 113 (3.3%) | ＜0.001 | | 1.2 | 0.96-1.50 | 0.103 |
|  | Weight loss | 429 (0.6%) | 106 (3.1%) | ＜0.001 | | 2.6 | 2.05-3.36 | *＜0.001* |

OR: Odds ratio, CI: Confidence interval
