# Supplementary material for: Risk factors for perioperative blood transfusion in patients undergoing total laparoscopic hysterectomy
Source: BMC Womens Health. 2024 Jan 24;24:65. doi: 10.1186/s12905-024-02908-4 (PMC10809697; doi:10.1186/s12905-024-02908-4)
Supplement: Supplementary file 4 — Additional file 4: Table S3. Relationship between blood transfusion and postoperative complications [file 12905_2024_2908_MOESM4_ESM.docx]

**Table S3** Relationship between blood transfusion and postoperative complications

| **Complications** | | **Univariate Analysis** | | | **Multivariate Logistic Regression** | | |
| --- | --- | --- | --- | --- | --- | --- | --- |
|  |  | **No transfusion** | **Transfusion** | **P** | **OR** | **95% CI** | **P** |
| **Medical complications** | |  |  |  |  |  |  |
|  | Sepsis | 301 (0.4%) | 93 (2.7%) | ＜0.001 | 2.4 | 1.80-3.26 | *＜0.001* |
|  | Acute myocardial infarction | 205 (0.3%) | 37 (1.1%) | ＜0.001 | 2.1 | 1.37-3.11 | *＜0.001* |
|  | Deep vein thrombosis | 161 (0.2%) | 59(1.7%) | ＜0.001 | 4.1 | 2.88-5.79 | *＜0.001* |
|  | Gastrointestinal hemorrhage | 44 (0.1%) | 32(0.9%) | ＜0.001 | 4.4 | 2.53-7.59 | *＜0.001* |
|  | Cardiac arrest | 38(0.1%) | 5 (0.1%) | 0.049 | 0.2 | 0.08-0.76 | 0.015 |
|  | Shock | 46 (0.1%) | 41(1.2%) | ＜0.001 | 6.6 | 4.03-10.70 | ＜0.001 |
|  | Pneumonia | 380 (0.5%) | 82 (2.4%) | ＜0.001 | 2.3 | 1.70-3.03 | *＜0.001* |
|  | Stroke | 136 (0.2%) | 17(0.5%) | ＜0.001 | 2.2 | 1.33-3.80 | *0.003* |
| **Surgical complications** | |  |  |  |  |  |  |
|  | Wound infection | 163 (0.2%) | 30 (0.9%) | ＜0.001 | 1.2 | 0.72-1.84 | 0.555 |
|  | Wound rupture | 68 (0.1%) | 12(0.3%) | ＜0.001 | 1.1 | 0.54-2.38 | 0.745 |
|  | Hemorrhage | 409(0.5%) | 273 (8.0%) | ＜0.001 | 12.3 | 10.45-14.58 | *＜0.001* |
|  | Pulmonary embolism | 184 (0.2%) | 54(1.6%) | ＜0.001 | 3.0 | 2.05-4.20 | *＜0.001* |
|  | Diseases of the genitourinary system | 2432 (3.2%) | 366 (10.7%) | ＜0.001 | 2.5 | 2.17-2.82 | *＜0.001* |

OR: Odds ratio, CI: Confidence interval
